# Family Planning as a Possible Measure to Alleviate Poverty in the Philippines – Beyond Sociocultural Norms and Pervasive Opposition

**DOI:** 10.15171/ijhpm.2017.57

**Published:** 2017-05-16

**Authors:** Akihiko Ozaki, Angeli Guadalupe, Arra Barrameda Saquido, Diana Francesca Gepte, Asaka Higuchi, Tomohiro Morita, Tetsuya Tanimoto

**Affiliations:** ^1^Department of Surgery, Minamisoma Municipal General Hospital, Fukushima, Japan.; ^2^Graduate School of Public Health, Teikyo University, Tokyo, Japan.; ^3^Department of Pediatrics, Brokenshire Memorial Hospital, Davao City, Philippines.; ^4^Department of Environmental and Occupational Health, University of the Philippines Manila, Malate Manila, Philippines.; ^5^College of Medicine, Pamantasan ng Lungsod ng Maynila, Metro Manila, Philippines.; ^6^Department of Hematology, Toranomon Hospital, Tokyo, Japan.; ^7^Department of Internal Medicine, Soma Central Hospital, Fukushima, Japan.; ^8^Department of Internal Medicine, Jyoban Hospital of Tokiwa Foundation, Fukushima, Japan.

## Dear Editor,

The 16th and current President of the Philippines, Rodrigo Duterte, aims to resolve poverty as a critical agenda. Although annual growth rate of the Philippine gross domestic product exceeded 5% since 2012, the estimated proportion of people living below the poverty threshold (2015) was 21.6% (22 million out of 100 million). Indeed, a significant economic disparity exists as demonstrated in its GINI index of 43.0 in 2013, a level higher than most Southeast Asian countries. Additionally, although the average total fertility rate in 2008 was 3.3 in the Philippines, its number reaches as high as 5.2 in the poorest household that is estimated to spend only around 2% of their total income on education.^1^ Actually, the children aged 6-17 in poorest households were less likely to attend school compared to those in richest households in 2011 (85.6% vs. 97.2%).^2^ As such, it has been hypothesized that there may be strong links among high fertility rate, poverty and limited opportunities of education in the country.

Given these speculations, President Duterte highlights the importance of population control using modern family planning measures in his war on poverty. However, the inadequate sexual health education and conservative sociocultural norms prove to be significant obstacles, as methods of contraception are widely disrespected by the general population. The Philippines is predominantly Catholic, whereby 80% of the local population subscribes to the teachings of the Church. Although constitutionally there is a separation of the Church and State, the Church remains influential in defining policies particularly in areas of reproductive health. Presently, abortion is prohibited in their constitution except for cases conducted to protect mothers’ health.

However, in reality, abortifacient agents are illegally sold in stalls adjacent to several Catholic cathedrals in Manila. Additionally, the lowest rate of condom use in Southeast Asia and increasing rate of casual sex contribute a considerable burden of sexually transmitted diseases, including human immunodeficiency virus (HIV).^3^ The reported number of HIV infections is currently 30 000, 80% of which have been newly registered since 2010.^3^ Further, the emerging risk of Zika virus infection or rubella would present a challenging situation to pregnant women.^4^

Under such conditions, there is a growing need that policymakers and public health professionals take into account the changing attitudes towards sexual intercourses among the general population in the country.

In 2012, Responsible Parenthood and Reproductive Health Act, also known as Reproductive Health Law, was enacted in the Philippines to improve an access to birth control measures after years of struggle.^5^ Although its constitutionality was questioned by the Supreme Court in 2013, the court finally declared that the law was constitutional in 2014.^5^ However, Temporary Restraining Order, which was subsequently issued by the Supreme Court, has restricted (1) the registration of new contraceptives and the re-registration of currently sold contraceptives when their permits expire, and (2) the purchase, distribution and use of subdermal implants by the Department of Health, eventually hampering the full implementation of the law until now.^6^ It is obvious that the current situation necessitates an early resolution, considering that there will be limited contraceptive measures available in the Philippines, such as tubal ligation, vasectomy and natural family planning methods, if the restraining order is not lifted and the current registration of contraceptives expire.^6^ We urge President Duterte to achieve a historic step to further improve healthcare in the Philippines – the full implementation of the Reproductive Health Law.

## Ethical issues

Not applicable.

## Competing interests

Authors declare that they have no competing interests.

## Authors’ contributions

AO wrote the manuscript. All authors conceptualized and designed the study, and revised the paper.

## Authors’ affiliations

^1^Department of Surgery, Minamisoma Municipal General Hospital, Fukushima, Japan. ^2^Graduate School of Public Health, Teikyo University, Tokyo, Japan. ^3^Department of Pediatrics, Brokenshire Memorial Hospital, Davao City, Philippines. ^4^Department of Environmental and Occupational Health, University of the Philippines Manila, Malate Manila, Philippines. ^5^College of Medicine, Pamantasan ng Lungsod ng Maynila, Metro Manila, Philippines. ^6^Department of Hematology, Toranomon Hospital, Tokyo, Japan. ^7^Department of Internal Medicine, Soma Central Hospital, Fukushima, Japan. ^8^Department of Internal Medicine, Jyoban Hospital of Tokiwa Foundation, Fukushima, Japan.
